# Enhancing Beta-Catenin Activity via GSK3beta Inhibition Protects PC12 Cells against Rotenone Toxicity through Nurr1 Induction

**DOI:** 10.1371/journal.pone.0152931

**Published:** 2016-04-05

**Authors:** Limin Zhang, Luan Cen, Shaogang Qu, Lei Wei, Mingshu Mo, Junmin Feng, Congcong Sun, Yousheng Xiao, Qin Luo, Shaomin Li, Xinling Yang, Pingyi Xu

**Affiliations:** 1 Department of Neurology, The First Affiliated Hospital of Sun Yat-sen University, Guangdong 510080, China; 2 Department of Neurology, The First Affiliated Hospital of Zhengzhou University, Zhengzhou, Henan Province, 450052, China; 3 Department of Blood Transfusion, The Fifth Affiliated Hospital, Southern Medical University, Guangzhou, Guangdong, 510900, China; 4 Department of Neurology, The Third Affiliated Hospital of Sun Yat-sen University, Guangdong, 510080, China; 5 Department of Neurology, Qilu Affiliated Hospital of Shandong University, Jinan, Shandong, 250001, China; 6 Department of Neurology, The Third Affiliated Hospital of Xinjiang Medical University, Urumqi, 830011, China; 7 Ann Romney Center for Neurologic Disease, Brigham and Women’s Hospital, Harvard Medical School, Boston, MA, 02115, United States of America; 8 Department of Neurology, The First Affiliated Hospital of Guangzhou Medical University, Guangdong, 510080, China; Institute of Health Science, CHINA

## Abstract

Parkinson’s disease (PD) is characterized by progressive degeneration of dopaminergic (DA) neurons in the substantial nigra pars compacta. Increasing evidence showed that Wnt/β-catenin pathway and the orphan nuclear receptor Nurr1 play crucial roles in the survival and functional maintenance of DA neurons in the midbrain and GSK-3β antagonists LiCl and SB216763 were used to activate Wnt/β-catenin pathway experimentally. However, the detail mechanism underlying the neuroprotection against apoptosis on DA neuron is still unclear and the interaction between Wnt/β-catenin and Nurr1 remains undisclosed. In this study, using cell biological assay we investigated the function of Wnt/β-catenin and its crosstalk with Nurr1 on the course of PC12 cell degeneration *in vitro*. Our data showed that PC12 cell viability was inhibited by rotenone, but attenuated by GSK-3β antagonists LiCl or SB216763. The activity of Wnt/β-catenin pathway was deregulated on exposure of rotenone in a concentration-dependent manner. After the interference of β-catenin with siRNA, LiCl or SB216763 failed to protect PC12 cells from apoptosis by the rotenone toxicity. Our data confirmed that Wnt/β-catenin signaling activated by LiCl or SB216763 enhanced Nurr1 expression to 2.75 ± 0.55 and 4.06 ± 0.41 folds respectively compared with control detected by real-time PCR and the interaction of β-catenin with Nurr1 was identified by co-immunoprecipitate analysis. In conclusion, the data suggested that Wnt/β-catenin and Nurr1 are crucial factors in the survival of DA neurons, and the activation of Wnt/β-catenin pathway exerts protective effects on DA neurons partly by mean of a co-active pattern with Nurr1. This finding may shed a light on the potential treatment of Parkinson disease.

## Introduction

Parkinson’s disease (PD) is characterized by progressive degeneration of dopaminergic (DA) neurons in the substantial nigra pars compacta (SNc) [1–2]. While host genetics account for < 10% of cases, environmental neurotoxins, such as 1-methyl-4-phenyl-1, 2, 3, 6-tetrahydropyridine (MPTP), rotenone, and 6-hydroxydopamine (6-OHDA), play a significant role on the disease onset and/or progression of PD [3–4]. However, the molecular mechanism underlying the degeneration of dopaminergic neurons remains to disclose.

Wnt and Nurr1 are important factors in the survival and functional maintenance of DA neurons in midbrain [5–12]. The Wnt pathway is a part of highly-conserved antocrine-paracrine signaling cascades [13–15], and the canonical Wnt pathway, known as Wnt/β-catenin signaling pathway, is an important signaling branch for cell survival. Studies have revealed that Wnt/β-catenin pathway was deregulated in cellular and animal models of toxin-induced PD [5,9,16–17] and the related genes express abnormally in DA neurons in midbrain of PD patients [18]. As for Nurr1, it is highly expressed in DA neurons for cell differentiation [19–20] and neonate Nurr1^-/-^ mice died after 24 hours without DA neurons in the midbrain[21]. The polymorphisms of Nurr1 have been reported to result in a marked decrease of Nurr1 transcription in lymphocytes of affected individuals with PD[22]. Meanwhile, reduction of Nurr1 in adult mice gives rise to the vulnerability of DA neurons to oxidative stress [23] and accumulation of α-synuclein[24]. However, so far what is the detail mechanism of Wnt/β-catenin interacting with Nurr1 to protect DA neurons from apoptosis remains unclear. In this study, using rotenone toxic cell model of PD we investigated the function of Wnt/β-catenin and Nurr1 against neurodegeneration and try to explore the mutual regulation related to their potential crosstalk in differentiated PC12 cells.

## Materials and Methods

### Cell Culture and Treatments

Differentiated PC12 cells, which were obtained 5 hours after the addition of NGF, were purchased from the Cell Library of the Chinese Academy of Science (Shanghai, China), maintained in Dulbecco's Modified Eagle's Medium (DMEM) with high glucose (Invitrogen, Carlsbad, California, USA) supplemented with 10% fetal bovine serum (FBS, Invitrogen, Carlsbad, California, USA), 100 U/mL benzyl penicillin, and 100 mg/L streptomycin (Gibco, Grand Island, NY, USA) and cultured in a humidified incubator with 5% CO_2_ at 37°C. Cells were seeded on 96-well plates, 6-well plates or 25cm^2^ plastic flasks at a density of 1×10^5^ cells/mL for 24 hours. The culture of HEK-293T cells was based on the instruction of Invitrogen.

To investigate Wnt/β-catenin signaling pathway, 2 selective GSK-3β inhibitors LiCl and SB216763 were used. LiCl (Sigma, St. Louis, MO, USA) was added into the cultures for 72 hours followed by rotenone treatment for 24 h. SB216763 (Sigma, St. Louis, MO, USA) was added into the cultures 30 minutes prior to rotenone 24hours-exposure (Sigma, St. Louis, MO, USA). In β-catenin gene silencing tests, differentiated PC12 cells were treated with the siRNA for 6 hours, and then the medium was replaced and added with LiCl for another 72 hours, followed by treatment with 1 μmol/L rotenone for last 24 hours. Similarly, following the siRNA protocol, differentiated PC12 cells were treated with SB216763 30 minutes prior to 24 hours of rotenone.

### Cell Counting Kit-8 Assay

The cell counting kit-8 (CCK-8) assay was performed to measure PC12 cells viability following rotenone, LiCl, and SB216763 treatments, respectively. Briefly, 10 μL of the CCK-8 kit reagent per 100 μL of medium was added to the wells of 96-well plate and incubated at 37°C for 2 hour. Cell viability was assessed by measuring the absorbance at 450nm with ELISA plate reader. The results were expressed as a percentage of the control group. Each treatment group was replicated in three wells.

### Caspase-3 Activity Assay

Caspase-3 activity was determined using the Caspase-3/CPP32 Fluor metric Assay Kit (Bio vision, USA). For each assay, 50 μg of cell lysate was loaded and read on the fluorescence microliter (Spectra Max Gemini EM, Molecular Devices, USA) with 400 nm excitation and 505 nm emission filter. Caspase-3 activity in sample was expressed as folds comparing to control.

### Gene Silencing with Small Interfering RNA (siRNA)

B-catenin siRNA or control siRNA were chemically synthesized by Guangzhou Riborio CO. LTD (Guangzhou, China). β-catenin siRNA: sense strand: 5’ -GCACCAUGCAGAAUACAAA dTdT -3’, Antisense strand: 5’ -UUUGUAUUCUGCAUGGUGCTdTd -3’. Cells were transfected with 100 nmol/L siRNA at 60% confluence using Lipofectamine2000 (Invitrogen, Carlsbad, CA, USA) according to the transfection protocol. The cells were harvested for analysis the transcription and protein of β-catenin at 24 h or 72 h following transfection, respectively. During the transfection, 2 mmol/L LiCl or 5 μmmol/L SB216763 was added to determine whether these chemicals protect PC12 cells from rotenone toxicity under depletion of β-catenin. At that time, total RNA was collected to detect the relative mRNA level of the target genes including β-catenin and Nurr1.

### RNA Extraction, Reverse Transcription and Real-Time PCR

Total RNA was isolated using TRIzol reagent (Invitrogen, Carlsbad, California, USA) and reverse transcribed into first-strand complementary DNA (cDNA) using reverse transcription kit (TaKaRa, Dalian, China). Real-time polymerase chain reaction (PCR) was performed in a fluorescent temperature cycler (ABI-Prism 7700 Sequence Detection System, Applied Biosystems) using a Light Cycler FastStart DNA Master Plus SYBR Green kit (Roche Diagnostics GmbH) with the following primes. β-catenin forward, 5’-GTCTGAGGACAAGCCACAGGACTAC-3’; β-catenin reverse, 5’- AATGTCCAGTCCGAGATCA GCA-3’; Nurr1 forward, 5’- CCAATCCGGCAATGACCAG-3’; Nurr1 reverse, 5’-TGATGATCTCCAT AGAGCCAGTCAG -3’. Rat GAPDH gene was used as an internal control. After 94°C for 4 min, the experimental reaction consisted of 39 cycles of 94°C for 15 s and 61°C for 45 s. Fluorescent readings from real-time PCR reactions were quantitatively analyzed by determining the Ct difference (delta Ct) between β-catenin/Nurr1 and the internal GAPDH control. The mRNA expression levels were determined by the formation of 2-delta Ct.

### Plasmid Construction and Co-Immunoprecipitation

Full-length human Nurr1 and β-catenin genes were inserted into pShuttle-IRES-hrGFP-2 vectors (Novagen, USA). HEK-293T cells or PC12 cells were transfected with vectors carrying Nurr1, β-catenin gene, or control vector with EndoFectin^TM^ reagent Plus (GeneCopoeia Inc., USA). At 72 h after transfection, cell lysates were prepared for co-immunoprecipitation.

A Pierce Immunoprecipitation Kit (Thermo Fisher Scientific, USA) was used to detect the immunoprecipitation of Nurr1 with β-catenin proteins in transfected HEK-293T cells or PC12 cells. Briefly, cells were lysed in ice-cold immunoprecipitation (IP) buffer containing 50 mmol/L Tris-HCl (pH 8.0), 150 mmol/L NaCl, 1% NP-40, 1 mmol/L Na_3_VO_4_, and protease inhibitor cocktail (Nacalai Tesque). The supernatant was pre-cleared with protein-A-Sepharose beads and incubated overnight at 4°C. After incubation, protein-A-Sepharose beads were added for 4 h to make the immune complex and washed in the washing buffer I (50 mM Tris-Cl pH7.6 with PI, 150 mM NaCl, 1% NP40, 0.5% Sodium deoxycholate, 4% protease inhibitor cocktail tablets), Washing buffer II (50 mM Tris-Cl pH7.6, 50 mM NaCl, 0.1% NP40, 0.05% Sodium deoxycholate), Washing buffer III (50 mM Tris-Cl pH7.6, 0.1% NP40, 0.05% Sodium deoxycholate, ddH2O) at 4°C. And then 500 μg of cell lysate was used to co-immunoprecipitation with rabbit anti-β-catenin (1:1,000 dilution, Cell Signaling Technology Inc., Danvers, MA, USA) and mouse Nurr1 antibodies (1:1,000 dilution, sigma, USA). Western blotting was carried out to detect Nurr1 and β-catenin respectively. Normal IgG (rabbit, Santa Cruz Biotechnology) was used as a control.

### Western Blotting

Immunoblotting was performed in accordance with a standard procedure. Briefly, cells were homogenized with ice-cold lysis buffer (50 mM Tris–HCl, 150 mM NaCl, 1 mM EDTA, and 0.5 mM Triton X-100, pH 7.4) containing protease inhibitor cocktail tablets (Roche, Basel, Switzerland). BCA protein assay kit (Thermo Fisher Scientific Inc., IL, USA) was used to measure protein concentrations. Both 20μg proteins and in each group and 5ug protein marker were separated by 10% SDS-PAGE and transferred to PVDF membranes (Millipore, USA). Positive and negative controls also were used for the first time to detect the specificity antibodies. After the membranes were blocked in 5% skim milk, 0.05% Tween 20, and Tris-buffered saline (TBS) for 1 hour, they were incubated with primary antibodies: rabbit anti-Wnt3a (1:1,000 dilution, 09–162, Millipore, USA), rabbit anti-β-catenin (1:200 dilution, Cell Signaling Technology Inc., 8480, Danvers, MA, USA), rabbit anti-non-phospho-β-catenin (1:200 dilution, Cell Signaling Technology Inc., 8814, Danvers, MA, USA), rabbit anti-Nurr1 (1:1,000 dilution, Sigma, N6413, USA) and rabbit anti-GAPDH (1:8,000 dilution, R&D, AF5718, USA) overnight at 4°C. The second horseradish peroxidase (HRP)-labeled antibody (1:3000 dilution, Cell Signaling Technology Inc., Danvers, MA, USA) were used in the next day. The bands were visualized using enhanced chemiluminescences (ECL) method (Millipore, USA). Membranes probed for GAPDH used as an internal control. The protein bands were quantified using image analysis software (Image J, V.1.42, National Institutes of Health, Bethesda, MD) and the protein levels were expressed as percent (%) of controls.

### GST Pull Down Assay

The GST fusion proteins used for GST pull down assay were produced by cloning Nurr1 into pGEX6p1 (Amersham Pharmacia Biotech, UK). The primers used for the clone were: AF1 for Nurr1 (Forward, 5’-CG CCTTGTGTTCAGGCGCAGTATG -3’. reverse, 5’- CTCGAGCTAGAAGAGTGAAAGGCGG GAGA C-3’), DBD for Nurr1 (Forward, 5’- CG GGCTCCCTTCA CAACTTCCAC -3’. reverse, 5’- CTC GAGCTAATCCTGTGGGCTCTTCGGTTT-3’), AF2 for Nurr1 (Forward, 5’-CGGGGCCCGAAAGG TAAGGTGTCCAGGAAA-3’. reverse, 5’-CTCGAGCTAAAACCGAAGAGCCCACAGGAT-3’). Nuclear proteins containing β-catenin were obtained by transfecting PC12 cells with β-catenin vectors for 24 hours and incubated with GST-fused mutants of Nurr1, AF1, DBD, AF2. And washed with Wash buffer containing 50mM Tris·HCl(pH7.3), 150mM NaCl, 3mM MgCl2, 1mM EDTA, 1mM DTT, 0.5%Triton X-100 β-catenin immobilized on glutathione-Sepharose beads prior to analysis by sodium dodecylsulfate-polyacrylamide gel electrophoresis.

### Chromatin Immunoprecipitation Sequence (ChIP-seq) Assay

Soluble chromatin from PC12 cells was prepared with an acetyl-histone H4 immunoprecipitation assay kit (Upstate Biotechnology) and immunoprecipitated with antibodies against β-catenin in the presence, absence of LiCl and β-catenin siRNA. Specific primer pairs were designed to amplify the promoter region of Nurr1 (Forward 5’-ACAATGTTTGGCTTCCTTGG-3’, reverse 5’- AACCACCAGCCTCCATACAC -3’). The CHIP resulting DNA samples were amplified by real time quantitative using SYBR Green master Mix (Applied Biosystems, Saint-Aubin, France).

## Statistical Analysis

All results were presented as means ± standard deviation (SD). One-way analysis of variance (ANOVA) followed by Student–Newman–Keuls test were used to compare differences between means in more than two groups. The level of significance was set at P < 0.05. All the statistical analyses were performed with SPSS 21.0 software for Windows (SPSS Inc., Chicago, IL, USA).

## Results

### Rotenone Induced Cell Injury and Deregulated Wnt/β-Catenin Pathway

**Fig 1:**
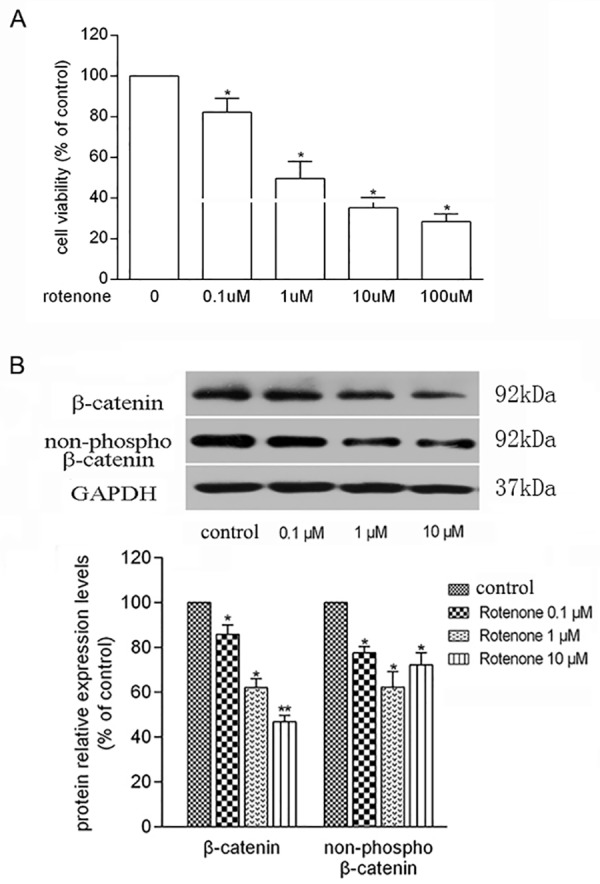
Rotenone affected the cell viability and Wnt/β-catenin of PC12 cells. A. cells were treated with 0~100 μmol/L (μM) rotenone for 24 hours. Cell viability was measured as percentage relative to vehicle (*P < 0.05; **P < 0.01). B. Rotenone deregulated Wnt3a, β-catenin, and non-phospho β-catenin in a concentration-dependent manner. The relative intensity of protein band was normalized to GAPDH by Image J software (*P < 0.05; **P < 0.01). All data presented as means ± SD from three independent experiments.

As showed in Fig 1A, treatment of 0.1~100 μmol/L rotenone for 24 hours caused a concentration-dependent reduction of PC12 cell viability. 1 μmol/L rotenone reduced the cell viability to nearly half (49.68 ± 8.24%) (Fig 1A), which was referenced to the subsequent experiments. Remarkable decreases were observed in the protein levels of β-catenin and non-phospho-β-catenin, molecules representing the activity of Wnt/β-catenin signaling, in a rotenone concentration-dependent manner (Fig 1B). Our results demonstrate that Wnt/β-catenin signaling was deregulated in the toxic cell model induced by rotenone.

### LiCl Attenuated Rotenone-Induced Cell Injury

**Fig 2:**
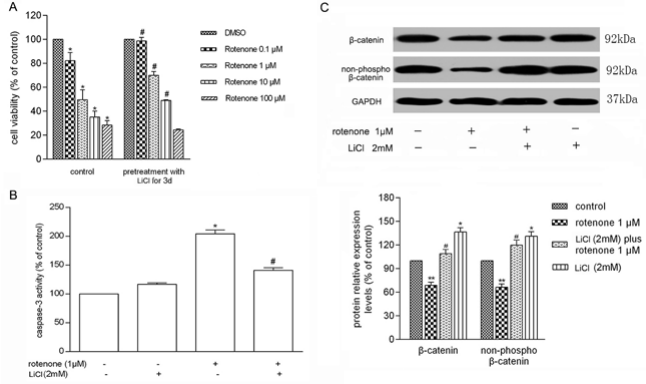
LiCl activated Wnt/β-catenin pathway for the survival of PC12 cells. PC12 Cells were pretreated with 2 mmol/L LiCl for 72 hours or 7 days prior to 1 μmol/L (μM) rotenone exposure, respectively. A. LiCl attenuated rotenone-induced cell injury. The cell viability was assessed as a percentage relative to vehicle group (n = 6 per group) and means ± SD (*P< 0.05; ^#^*P*< 0.01 compared to equivalent rotenone treated group). B. LiCl inhibited caspase-3 activity in PC12 cells (*P< 0.05; ^#^*P*< 0.05 compared to rotenone treated group). C. LiCl reversed the down-regulation of Wnt3a, β-catenin and non-phospho-β-catenin induced by rotenone (*P < 0.05; **P < 0.01 compared to vehicle; ^#^*P* < 0.05 compared to rotenone treated group). All data are presented as means ± SD from three independent experiments.

We further ask whether interfering of Wnt/β-catenin signaling would affect the outcome of rotenone-treated cells. Pretreatment of 2 mM LiCl for 72h attenuated rotenone-induced cell injury when the concentration of rotenone was in the range of 0.1~10μM (Fig 2A). Consistently, using Caspase-3/CPP32 Fluor metric Assay Kit, the caspase-3 activity was detected significantly up to 204.51 ± 6.44% in PC12 cells treated with 1 μmol/L rotenone compared with non-treated control (Fig 2B). However, when LiCl was pre-added 72 hours before rotenone, the activity of caspase-3 was down to 141.09 ± 4.67% (*P* < 0.01) in comparison to rotenone treatment alone. To further investigate Wnt/β-catenin signaling in rotenone-induced cell injury, we examined Wnt3a, β-catenin and non-phospho-β-catenin by Western blotting. After the cells were exposed to 1 μmol/L rotenone, a significant decrease in β-catenin and non-phospho-β-catenin was found in PC12 cells (68.89 ± 3.84%, 66.39 ± 4.14% respectively, *P* < 0.05). Pretreatment of 2 mmol/L LiCl significantly attenuated those changed (Fig 2C). Collectively, our data here demonstrate that the activation of Wnt/β-catenin signaling by LiCl functions as a rescue of rotenone-induced cell injury.

### SB216763 Activated Wnt/β-Catenin and Attenuated Rotenone-Induced Cell Injury

**Fig 3:**
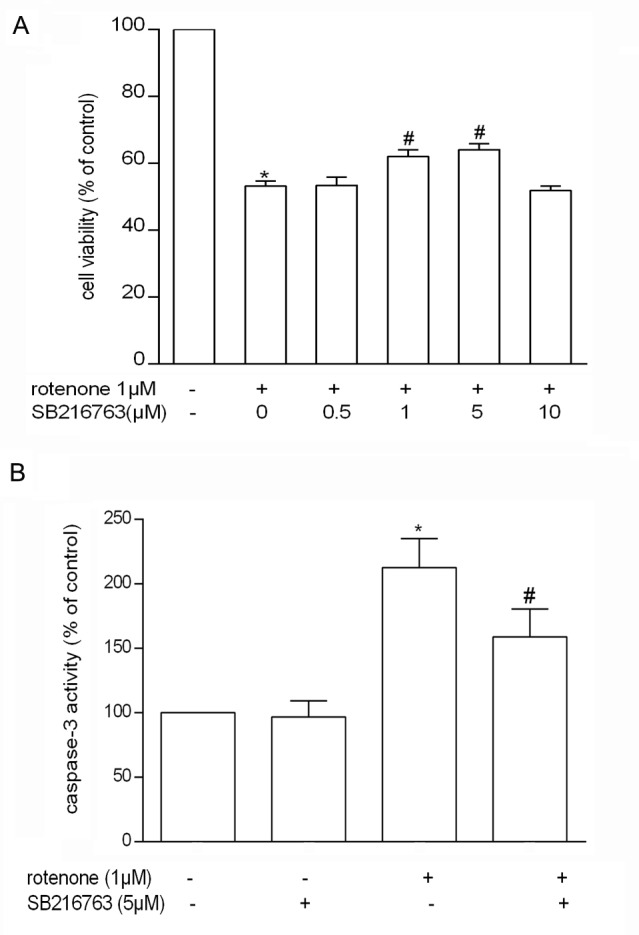
SB216763 activated Wnt/β-catenin pathway for the survival of PC12 cells. A. SB216763 attenuated rotenone-induced cell injury (*P< 0.05 compared to vehicle; ^#^, *P* < 0.05 compared to rotenone). B. SB216763 inhibited the caspase-3 activity. Cells pretreated with 0~10 μM SB216763 in 30 minutes prior to 24 hours treatment of 1 μM rotenone. Caspase-3 activity was assessed by Caspase-3/CPP32 Fluor metric Assay Kit, and expressed as percentages relative to vehicle (n = 6 per group) (* *P* < 0.05; ^#^*P* < 0.05 compared to rotenone treated group).

Similarly, GSK3β antagonist SB216763 was used to activate Wnt/β-catenin pathway to see its effect on rotenone-induced cell injury. Our data showed that SB216763 (0.5 to 10μM) attenuate rotenone-induced cell injury in a concentration-dependent manner (Fig 3A). Therefore 5 μmol/L SB216763 was used to the follow-up experiments for its protective effect on cell viability. Further experiment showed that 5 μmol/L SB216763 was sufficient to reverse the rotenone-increased caspase-3 activity from 212.63 ± 22.58% to 158.79 ± 21.64% (*P* < 0.05) (Fig 3B). Combined with LiCl result, we conclude that Wnt/β-catenin pathway was deregulated by rotenone treatment, and re-activation of it might alleviate rotenone-induced cell injury.

### β-Catenin Protected PC12 Cells from Rotenone-Induced Injury

**Fig 4:**
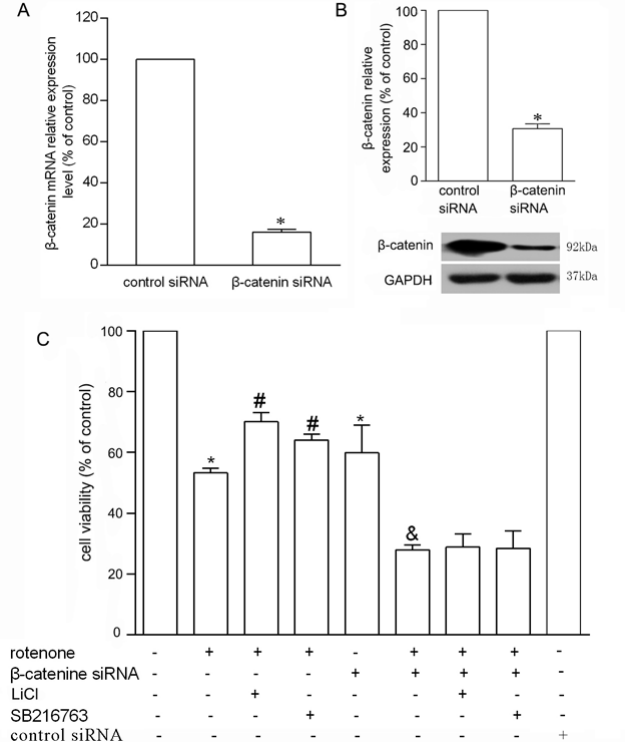
β-catenin prevented PC12 cells from death induced by rotenone. A. Real-time PCR for measuring the mRNA level of β-catenin which was down-regulated by siRNA (*P < 0.05). B. the protein level of β-catenin was inhibited in PC12 cells transfected with β-catenin siRNA (*P < 0.05). C. Under transfection of β-catenin siRNA, pretreatment with LiCl or SB216763 failed to attenuate the cell loss induced by rotenone. (*P< 0.05 compared to control, ^#^*P*< 0.05 compared to rotenone. ^&^*P*< 0.05 compared to β-catenin siRNA).

To look into the association between β-catenin and rotenone induced cell death, a siRNA strategy was used to knock down β-catenin gene. As shown in Fig 4, when PC12 cells were transfected with 100 nmol/L β-catenin siRNA, the level of β-catenin mRNA was reduced to 16.15 ± 1.36% within 24 hours which was detected by real-time PCR while the protein level was down to 30.79 ± 2.81% after 72 hours by Western blotting. PC12 cell viability was also significantly reduced to 59.85 ± 9.13% with the interference of 100 nmol/L β-catenin siRNA, while the cell viability was further worsened to 27.96 ± 1.59% by the treatment of β-catenin siRNA plus 1 μmol/L rotenone. Under the β-catenin siRNA condition, pretreatment with 2 mmol/L LiCl or 5 μmol/L SB216763 for 72 hours failed to attenuate the cell loss induced by rotenone and the cell viability were dropped to 28.83 ± 4.35% and 28.42 ± 5.73% respectively, which showed no significant difference compared with 1 μmol/L rotenone only (*P* > 0.05) (Fig 4C).

### The Regulation of Wnt/β-Catenin on Nurr1 Expression in PC12 Cells

**Fig 5:**
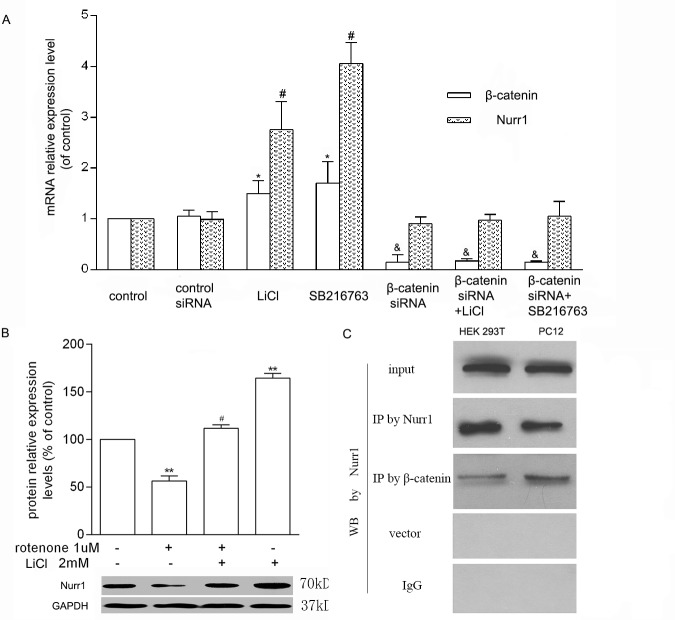
Crosstalk between β-catenin and Nurr1. A. LiCl or SB216763 activated the Wnt/β-catenin to induce Nurr1 transcription, while β-catenin siRNA had no effect on Nurr1 expression, which was detected by Real-time PCR (*P< 0.05 β-catenin compared to control; ^#^*P* < 0.05 Nurr1 compared to control). B. LiCl reversed the down-regulation of Nurr1 expression induced by rotenone *in vitro*. The relative band intensities of Nurr1 were normalized to GAPDH (**P < 0.01 compared to control; ^#^ *P* < 0.05 compared to rotenone). C. Interaction of Nurr1 with β-catenin. Cells were transfected with plasmids carrying Nurr1 or β-catenin gene respectively. Collected lysates from HEK 293T and PC12 were subjected to co-immunoprecipitation by Nurr1 or β-catenin antibodies to detect their crosstalk.

Previous studies have demonstrated that both β-catenin and Nurr1 have protective effects on DA neurons. But little is known about whether there is a synergistic effect between β-catenin and Nurr1 and whether one regulates the expression of the other. So the interaction between β-catenin and Nurr1 was investigated. As our results showed, LiCl or SB216763 both significantly raised β-catenin and Nurr1 mRNA levels (Fig 5A). Similarly, the data of western blotting showed that treatment of rotenone caused a significant decrease of Nurr1 level, while pretreatment of LiCl reversed this change (Fig 5B). The co-immunoprecipitation of Nurr1 with β-catenin was detected in PC12, or HEK 293T cells which were transfected with Nurr1 or β-catenin plasmids, suggesting that Nurr1 and β-catenin were interacted with each other in the transfected cells (Fig 5C). To summary, our data suggest that β-catenin might directly interact with Nurr1 to execute its function of cell survival against rotenone.

### The Analysis of the Interaction between β-Catenin and Nurr1

**Fig 6:**
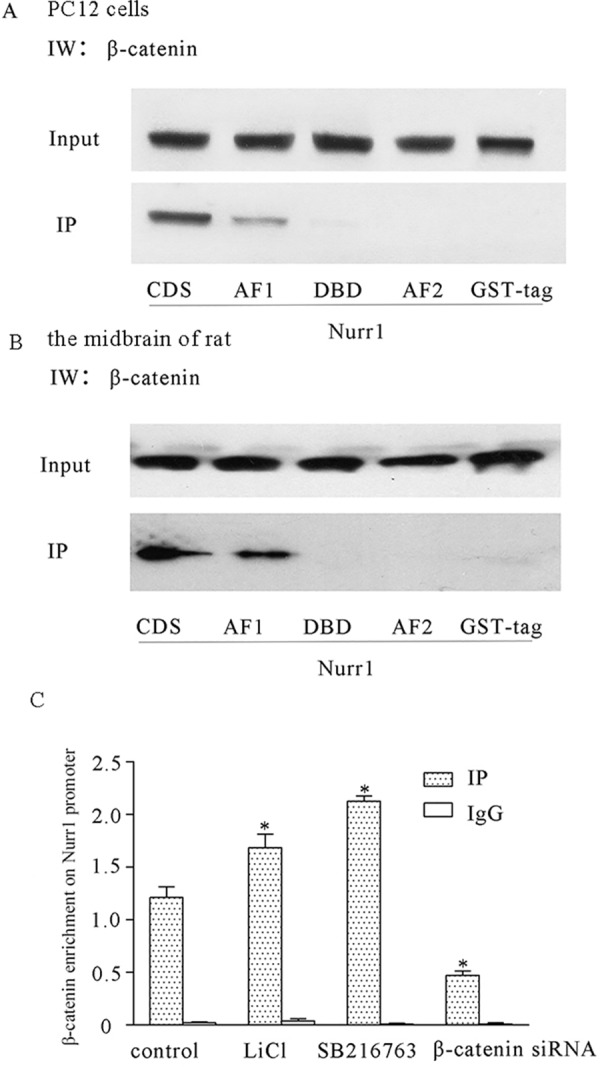
The interaction of β-catenin with Nurr1. The binding site was at AF1, the domain of Nurr1 with β-catenin detected by GST pull down assay. A. PC12 cells. B. Midbrain of rat. C. β-catenin bonded to the up-stream promoter of Nurr1, resulting in enhanced transcription of Nurr1 (*P < 0.05 compared to control).

The crosstalk between β-catenin with Nurr1 was further studied based on the hypothesis above in both PC12 cells and rat midbrains. As shown in Fig 6A and 6B, in PC12 cells and the midbrain of rat, the interaction between β-catenin and the AF1 domain of Nurr1 was detected by the GST pull down assay. Chromatin immunoprecipitation assay also confirmed that β-catenin directly bound to the promoter region of Nurr1 gene (Fig 6C). GSK3β inhibitor LiCl and SB216763 enhanced the bindings of β-catenin to 1.686±0.13 and 2.128±0.05 respectively compared with control level: 1.213±0.10. Consistent with previous result, knockdown of β-catenin with siRNA decreased the enrichment level of β-catenin on the promoter region of Nurr1 down to 0.473±0.04 (*P* < 0.05). These data clearly showed that mechanistically β-catenin not only directly regulates the gene expression of Nurr1 but also interacts with Nurr1 protein to execute their cell survival function against rotenone.

## Discussion

This study demonstrated the protection effect of Wnt/β-catenin and Nurr1 on the differentiated PC12 cells against rotenone-induced DA neurotoxin. Differentiated PC12 cells are widely used as a suitable model to explore the mechanisms of degeneration of DA neuron in Parkinson’s disease [25] and the function of the Wnt/β-catenin signaling pathway[26–28]. Here we confirmed that rotenone caused a concentration-dependent reduction in the cell viability, and 50% of the cell viability affected by 1 μmol/L rotenone was set as a desired cellular model for follow-up tests. We also identified that β-catenin and non-phospho β-catenin of Wnt/β-catenin signaling pathway were expressed in differentiated PC12 cells. As an environment factor, rotenone selectively inhibits mitochondrial complex I, causing mitochondrial impairment via oxidative stress, and resulting in an increase of apoptotic markers of neurons such as caspase-3 activity, caspase-9 activity, Bax expression, cytochrome c, lactate dehydrogenase release and microtubule destabilization in rotenone-evoked Parkinsonism [29–34]. In our experiment, rotenone was demonstrated to increase caspase-3 activity, reduce the PC12 cell viability, deregulate the activity of Wnt/β-catenin signaling pathway in a concentration-dependent manner. The relative level of Wnt3a, β-catenin and non-phospho-β-catenin was found at a similar reduction pattern. Similarly many reports showed that MPTP/MPP^+^ or 6-OHDA resulted in dysfunction of Wnt/β-catenin signaling pathway both in mice and cellular models of Parkinson disease [30, 35–36].

Our data indicated that Wnt/β-catenin signaling pathway can be activated by GSK3β antagonists LiCl or SB216763 in PC12 cells. LiCl or SB216763 was found to increase the level of β-catenin and non-phospho-β-catenin (a form of active β-catenin) and attenuate the cell death affected by rotenone, but either of them failed to protect β-catenin siRNA transfected PC12 cells from the cell death induced by rotenone, indicating β-catenin might be a key molecule in Wnt/β-catenin pathway for PC12 cells to survive. Consistently, our previous data showed that 6-OHDA decreased β-catenin level, but activation of Wnt/β-catenin pathway by exogenous wnt1 attenuated 6-OHDA-induced neurotoxicity through restoring mitochondria and endoplasmic reticulum function [30]. In addition, activation of the Wnt/β-catenin signaling pathway by exogenous wnt1 was reported to exert robust neuroprotection on the caspase-3 activation. Besides, canonical Wnt/β-catenin pathway is required for the survival of adult DA neurons in aged mice following MPTP insult [9], and this pathway was demonstrated to play a critical role in promoting neuronal survival in Parkinson disease and Alzheimer disease [37–44]. In summary, our data provide one more piece of solid evidence on the critical survival effect of Wnt/β-catenin pathway for DA neuronal toxin resistance.

The mechanisms underlying the protection of the Wnt/β-catenin signaling pathway on DA neurons remain elusive. It involves in multiple mechanisms including the regulation of pro-survival process or anti-apoptosis [5,9], plasticity of neuroprogenitors of subventricular zone in adult brain [35], enhanced DA differentiation potential of multipotent clonogenic neural stem/progenitor cells (mNPCs) in the adult midbrain aqueduct periventricular regions (Aq-PVRs) [45], synaptic plasticity [46–47], inhibition of oxidative stress and inflammation[30,45], and so on. In this study, we demonstrated the interaction between β-catenin and AF1 region of Nurr1 as the underlying mechanism. Furthermore, using CHIP PCR assay, we found that LiCl or SB216763 promoted β-catenin binding on the upstream promoter region of Nurr1 and increased the transcription of Nurr1. Thus we hypothesis that as a downstream of Wnt/β-catenin signaling pathway, β-catenin not only directly regulates Nurr1 gene expression but also interacts with Nurr1 protein to improve the anti-apoptosis capability of PC 12 cells and dopaminergic neurons. In agreement with our discovery, Wnt/β-catenin signaling pathway was reported to enhance the transcription of Nurr1 in HEK293 cells [48] and Nurr1 mRNA level was markedly increased in BATGAL mice and 10-month-old △Cat mice, two models of constitutive β-catenin activation mice[45,49]. Moreover, β-catenin activated by BIO, a known inhibitor of GSK3β, was reported to increase production of aldosterone by stimulating Nurr1 expression in mouse Y1 adrenocortical cells in vitro [49]. In HEK293T cells, β-catenin facilitated Nurr1 mediated transactivation of the NBRE3tk-LUC reporter regulated by three binding sites for the NR4A receptors. Furthermore, knocking down β-catenin resulted in a significant decrease in both Nurr1 mRNA and protein in H295R cells [49]. However, in U2-OS and HeLa cells, β-catenin was reported to repress Nurr1 mediated transactivation of the NBRE3tk-LUC reporter [48]. These inconsistent observations suggested that Nurr1 expression regulated by β-catenin may vary with different cell types. In short, β-catenin inducing Nurr1 expression or Nurr1 affecting the activity of β-catenin suggests that a positive regulatory loop may exist between β-catenin (activation of Nurr1) and Nurr1 itself (reciprocal inhibition of β-catenin). For our results, it should be kept in mind that although differentiated PC12 cells adopt a neuronal-like phenotype, the cells might not completely represent typical DA neurons[50–52]. Further studies need to be done in animal models of PD. In conclusion, our data supported that the enhancement of Wnt/β-catenin signaling pathway protect dopaminergic cells against rotenone-toxicity. In term of mechanism, Wnt/β-catenin signaling play a role in restoring mitochondria and endoplasmic reticulum function by interacting partly with Nurr1 on its dopaminergic regulation effects. In addition to the well-developed role for Wnt signaling during CNS development of the VM dopaminergic system, it is impressive that Wnt signaling could, in part, affect the activity of Nurr1 in the maintenance of dopaminergic neurons function.
